# Extirpation of Rectum

**Published:** 1873-11

**Authors:** William Ashbridge


Extirpation of the Rectum.—The extirpation of the rectum, with the formation of a musculo-cutaneous perineal flap, according to Hueter, is a method by which the difficulties and dangers incident to the operation as usually performed are avoided. The usual dangers, as enumerated by him, are the following four: hemorrhage from the hemorrhoidal arteries which are necessarily cut, the difficulty of their ligation, possible injury to the peritoneum, finally, incontinence of fæces.

The first act in the operation consists in the formation of a tongue-shaped flap, with the free convex end looking towards the scrotum, the base being bounded by a line joining the tubera ischii. The sphincter ani must next be separated, and the muscular tissue and mucous membrane of the rectum cut obliquely through at a point one centimetre above the anus, so that the entire ring of the anal opening is contained in the flap, and this is then thrown back. The bleeding vessels having been ligated, the extirpation of the gut is to be proceeded with. The edges of the wound are to be united with sutures—those in the back part of the wound being first applied—and the flap is then to be drawn into its old place and fastened by stitches. The after-treatment is made much easier by the fact that the fæces traverse the normal canal and do not soil the edges of the wound. This method of operating was tried in two cases: the first patient was a drunkard, who, after the operation, was attacked with delirium-tremens, and finally died of pneumonia; the second recovered without any bad symptoms, and left his bed two weeks after the operation. WILLIAM ASHBRIDGE, M.D.

HARVEY.—John Aubrey, who was at Harvey's funeral and "helpt to carry him into the vault," writes, "I have heard him [Harvey] say, that after his booke of the Circulation of the Blood came out, he fell mightily in his practice, and 'twas believed by the vulgar that he was crack-brained; and all the physicians were against his opinion, and enoyed him. All his profession would allow him to be an excellent anatomist, but I never heard of any that admired his therapeutique way. I knew several practitioners in this town [London] that would not have given 3*d.* for one of his bills, [prescriptions], and that a man could hardly tell by one of his bills what he did aim at."

